# Supplementary material for: SIRT5-dependent regulation of ASL controls arginine metabolism and T cell function
Source: bioRxiv. 2026 Jun 29:2026.06.24.734337. Preprint. [Version 1] doi: 10.64898/2026.06.24.734337 (PMC13345334; doi:10.64898/2026.06.24.734337)
Supplement: Supplement 1 [file media-1.pdf]

SUPPLEMENTAL INFORMATION

SIRT5-dependent regulation of ASL controls arginine metabolism and T cell function

Kim Han,[1] Rachael J. Klein,[1] Kaiyuan Wu,[2] Anna Chiara Russo,[1] Allison M. Meadows,[1] Thomas C. Recupero,[1] Rahul Sharma,[1] Anand K. Gupta,[1] Nathan T. Brandes,[3] Benjamin Schwarz,[3] and Michael N. Sack[1,4,*]

[1]Laboratory of Mitochondrial Biology and Metabolism, NHLBI, NIH, Bethesda, MD, USA

[2]Laboratory of Obesity and Metabolic Diseases, NHLBI, NIH, Bethesda, MD, USA

[3]Research Technologies Branch, NIAID, NIH, Hamilton, MT, USA

[4]Cardiovascular Branch, NHLBI, NIH, Bethesda, MD, USA

*Correspondence:

Michael N. Sack, Laboratory of Mitochondrial Biology and Metabolism, NHLBI, NIH,

Bldg. 10-CRC, Room 5-3342, 10 Center Drive, Bethesda, MD 20892, USA

Email: sackm@nih.gov

SUPPLEMENTAL MATERIALS

- **Supplemental Figures and Legends (4)**
- **Supplemental Table (1)**
- **Supplemental Datasets (2) - Uploaded separately as Excel files**

  Supplemental Dataset 1: Integrated multi-omics dataset (lipidomics, metabolomics, and RNA-seq), related to Figure 1

  Supplemental Dataset 2: Metabolomics dataset of SIRT5-dependent metabolic remodeling, related to Figure 5

Supplemental Figures and Legends

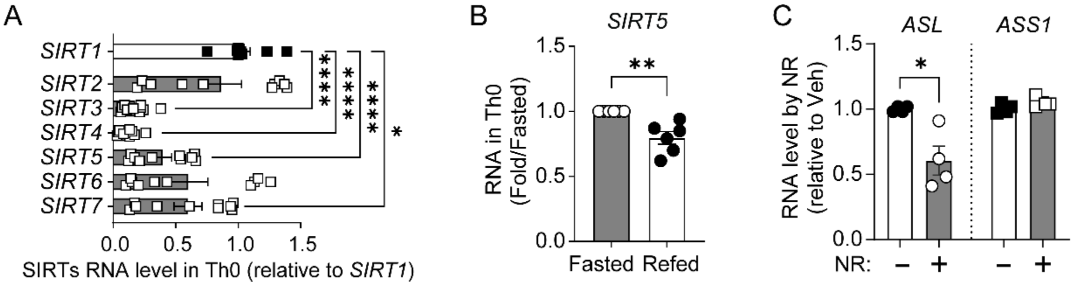

Supplementary Figure 1. Expression of sirtuins and arginine pathway genes in CD4⁺ T cells. (**A**) Relative RNA expression of sirtuin family members (*SIRT1-7*) in $CD4^+$ T cells under Th0 conditions, normalized to *SIRT1*. (**B**) Relative expression of *SIRT5* in $CD4^+$ T cells under fasting and refeeding conditions. (**C**) Relative RNA expression of *ASL* and *ASS1* in $CD4^+$ T cells treated with NR compared to vehicle. Data are presented as mean ± SEM from independent biological replicates, with each dot representing an individual donor. Statistical significance was determined using one-way ANOVA with Šídák's multiple comparisons test (A) or paired two-tailed Student's *t*-test (B-C). *$p < 0.05$, **$p < 0.01$, ***$p < 0.001$.

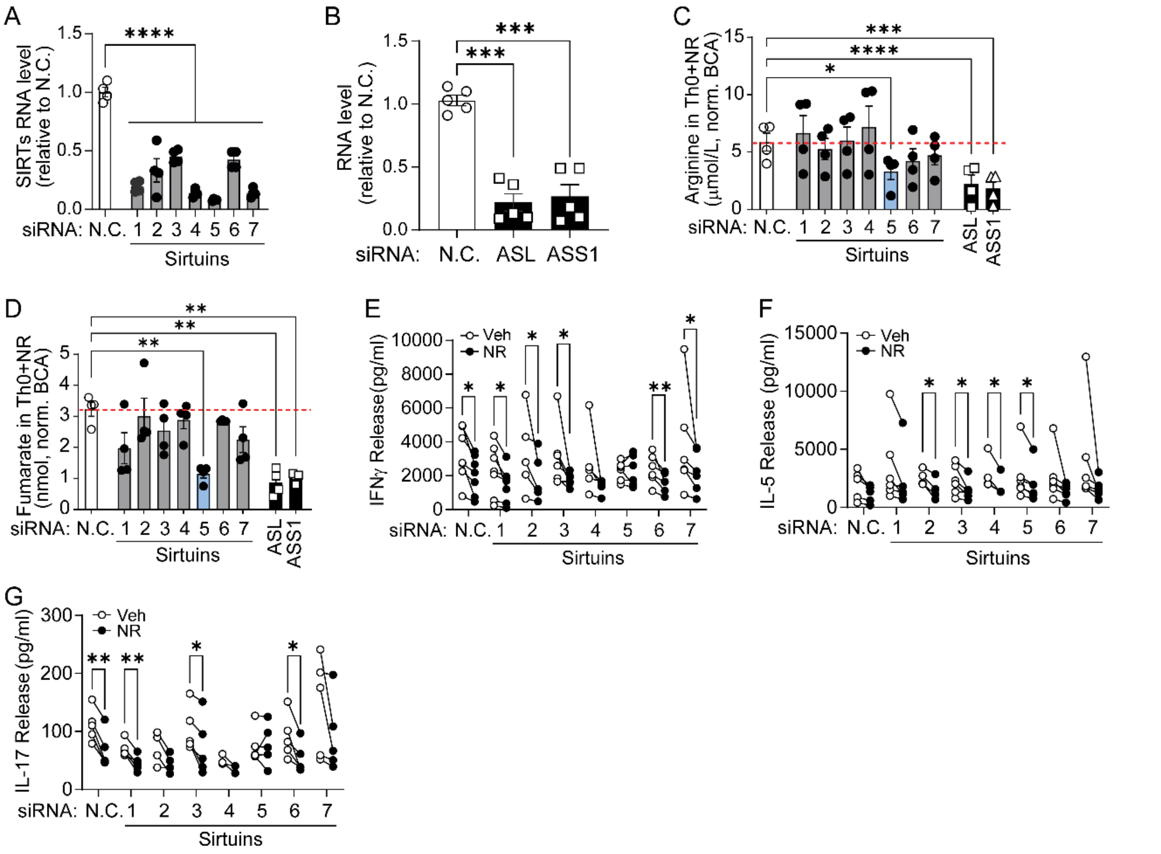

Supplementary Figure 2. Sirtuin screening identifies SIRT5 as a regulator of arginine metabolism in CD4⁺ T cells. (**A**) Relative RNA expression of sirtuins following siRNA-mediated knockdown, normalized to non-targeting control (N.C.). (**B**) Relative RNA expression of *ASL* and *ASS1* following siRNA-mediated knockdown. (**C-D**) Intracellular arginine and fumarate levels in $CD4^+$ T cells transfected with siRNAs targeting individual sirtuins (SIRT1-7), ASL, or ASS1 in the presence of NR. (**E-G**) Cytokine production (IFNγ, IL-5, and IL-17) in $CD4^+$ T cells transfected with siRNAs targeting individual sirtuins and treated with vehicle or NR. Data are presented as mean ± SEM from independent biological replicates, with each dot representing an individual donor. Statistical significance was determined using one-way ANOVA with Šídák's multiple comparisons test (A-D) or two-way ANOVA with Šídák's multiple comparisons test (E-G). $*p < 0.05$, $**p < 0.01$, $***p < 0.001$.

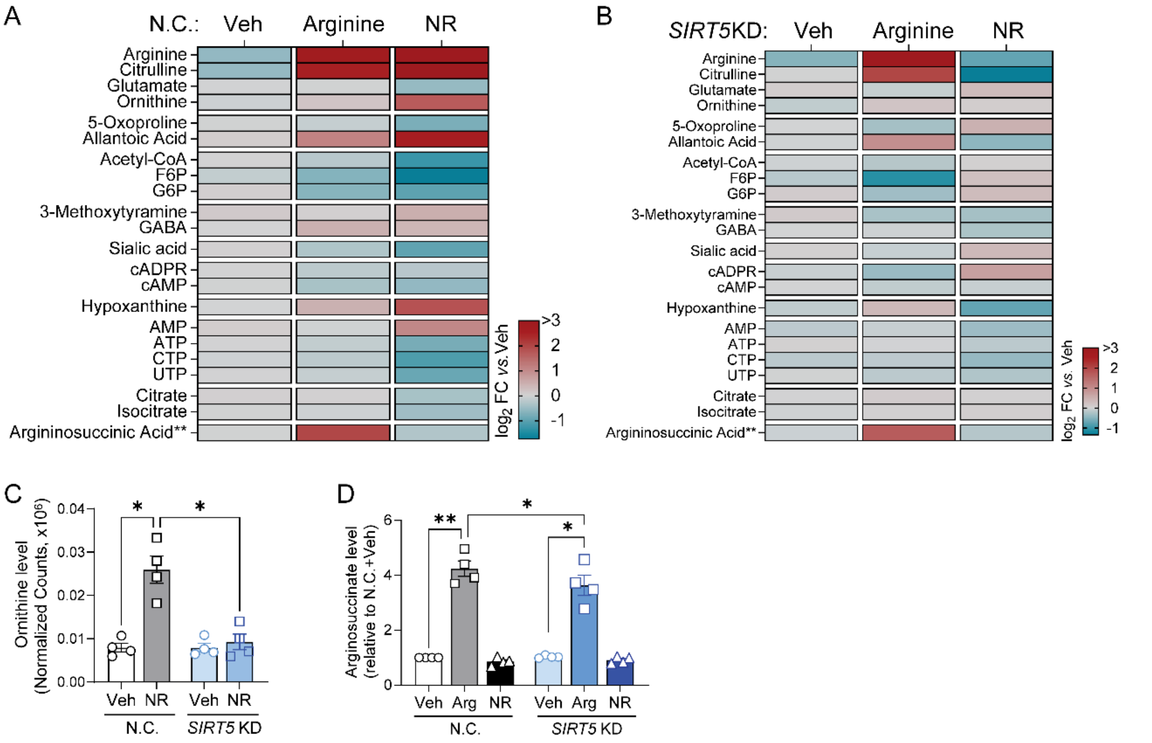

Supplementary Figure 3. Metabolomic responses to NR and arginine in control and SIRT5-dependent CD4$^+$ T cells. (A-B) Heatmaps showing metabolite abundance in non-targeting control (N.C., **A**) and SIRT5 knockdown (KD, **B**) CD4$^+$ T cells treated with vehicle, arginine, or NR. Data are presented as $\log_2$ fold change relative to vehicle-treated controls. Data are presented as $\log_2$ fold change relative to vehicle-treated controls. **(C)** Intracellular ornithine levels in N.C. and SIRT5 KD CD4$^+$ T cells treated with vehicle or NR. **(D)** Relative arginosuccinate levels in N.C. and SIRT5 KD CD4$^+$ T cells treated with vehicle, arginine, or NR. Arginosuccinate abundance was normalized to the N.C. vehicle condition. Data are presented as mean ± SEM from independent biological replicates, with each symbol representing an individual donor. Statistical significance was determined using paired two-tailed Student's *t*-test or two-way ANOVA with Šídák's multiple comparisons test, as appropriate. *$p < 0.05$, **$p < 0.01$.

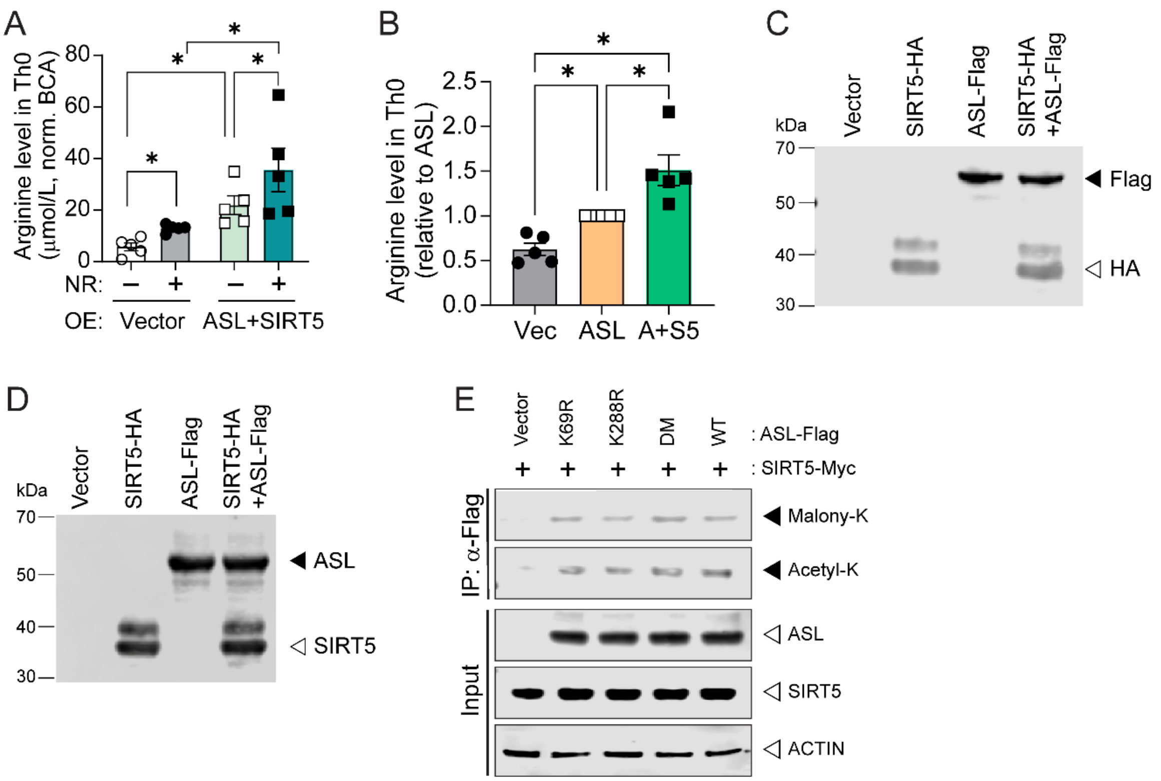

Supplementary Figure 4. Overexpression of SIRT5 and ASL regulates arginine metabolism and ASL acylation. (**A**) Intracellular arginine levels in CD4$^+$ T cells overexpressing ASL and SIRT5 in the presence or absence of NR. (**B-C**) Immunoblot analysis confirming expression of SIRT5-HA and ASL-Flag constructs used for co-immunoprecipitation studies. (**D**) ASL malonylation (Malonyl-K) and acetylation (Acetyl-K) assessed by Flag-IP and followed by immunoblotting in wild-type (WT), K69R, K288R, and double mutant (DM) in the presence of SIRT5. Data in (A-B) are presented as mean ± SEM from independent biological replicates. Statistical significance was determined using two-way ANOVA followed by Šídák's multiple comparisons test. *$p < 0.05$.

Supplemental Table 1. Pre-made primer sets used for quantitative real-time RT-PCR.

Oligonucleotides	SOURCE	IDENTIFIER
SIRT1	Qiagen	QT00051261
SIRT2	Qiagen	QT00069531
SIRT3	Qiagen	QT00091490
SIRT4	Qiagen	QT00202503
SIRT5	Qiagen	QT00047537
SIRT6	Qiagen	QT00056812
SIRT7	Qiagen	QT01020663
ASL	Qiagen	QT00054579
ASS1	Qiagen	QT00036456
RRN18S	Qiagen	QT00199367
b-ACTIN	Qiagen	QT00095431
